# Myocardial fibrosis as the first sign of cardiac involvement in a male patient with Fabry disease: report of a clinical case and discussion on the utility of the magnetic resonance in Fabry pathology

**DOI:** 10.1186/1471-2261-14-86

**Published:** 2014-07-16

**Authors:** Annalisa Sechi, Gaetano Nucifora, Gianluca Piccoli, Andrea Dardis, Bruno Bembi

**Affiliations:** 1 Regional Coordinator Centre for Rare Diseases, University Hospital Santa Maria della Misericordia, Udine, Italy; 2 Cardiothoracic Department, University Hospital Santa Maria della Misericordia, Udine, Italy; 3 Department of Diagnostic Imaging, University Hospital Santa Maria della Misericordia, Udine, Italy

**Keywords:** Fabry disease, Cardiovascular magnetic resonance, Myocardial fibrosis, Late gadolinium enhancement

## Abstract

**Background:**

Cardiovascular magnetic resonance (CMR) with late gadolinium enhancement (LGE) imaging is increasingly used to assess myocardial involvement in patients with Fabry disease, an X linked lipid storage disorder. However, it is often proposed as an optional tool. A different cardiomyopathic disease progression between male and female patients was hypothesised in previous studies, as in female myocardial fibrosis was found without left ventricular (LV) hypertrophy, while myocardial fibrosis was always detected in association to LV hypertrophy in men.

**Case presentation:**

A male Caucasian patient, 19 years old, diagnosed through a family-based molecular screening, presented with LGE of the LV inferolateral wall evidenced at the CMR, without LV hypertrophy, or other clinical signs of the disease.

**Conclusion:**

This is the first report of cardiac fibrosis as the first sign of organ involvement in a male patient with Fabry disease. This finding stresses the importance of performing CMR with LGE imaging for the initial staging and monitoring of Fabry patients of both genders.

## Background

Fabry disease is an X linked lysosomal storage disorder, characterised by the deficient activity of the enzyme α-galactosidase A, involved in the catabolism of glycosphingolipids. This enzymatic defect results in the accumulation of globotrialosylceramide within the cells, particularly in cardiomyocytes, kidney, neural and vascular endothelial cells, causing a progressive systemic disease, which can lead to severe cardiac, renal, and cerebrovascular complications [1]. Heterozygous females may become as severely affected as males, although generally at a later age [2]. In males the diagnosis is made by the demonstration of α-gal deficiency in leukocytes and/or by the identification of pathogenic mutations in the α-galactosidase gene (*GLA*, Xq22.1 region). In heterozygous females, the enzymatic activity is not a reliable test, due to random X-chromosome inactivation, rendering DNA sequencing of the *GLA* gene the only reliable test for the diagnosis confirmation in females. Once the GLA mutation is identified, a screening within the family is indicated in order to detect Fabry patients at an early stage [3], when the disease seems to be more responsive to the specific enzyme replacement therapy (ERT) [4,5].

Cardiac involvement in Fabry disease includes arrhythmias, ischemia, and cardiomyopathy and is associated with increased morbidity and mortality in both male and female patients [6]. Left ventricular (LV) hypertrophy is considered the most common cardiologic finding [1]. Studies using cardiovascular magnetic resonance (CMR), demonstrated that myocardial fibrosis, detected with late gadolinium enhancement (LGE), is also part of the natural history of Fabry disease [7,8]. A recent cardiovascular imaging research on patients with Fabry disease, showed a different cardiomyopathic involvement between male and female subjects: myocardial fibrosis was always detected in association to LV hypertrophy in men, while in females LGE was found even with normal LV thickness [9].

As far as we know, this is the first report of cardiac fibrosis as the first sign of organ involvement in a male patient with Fabry disease.

## Case presentation

**Figure 1:**
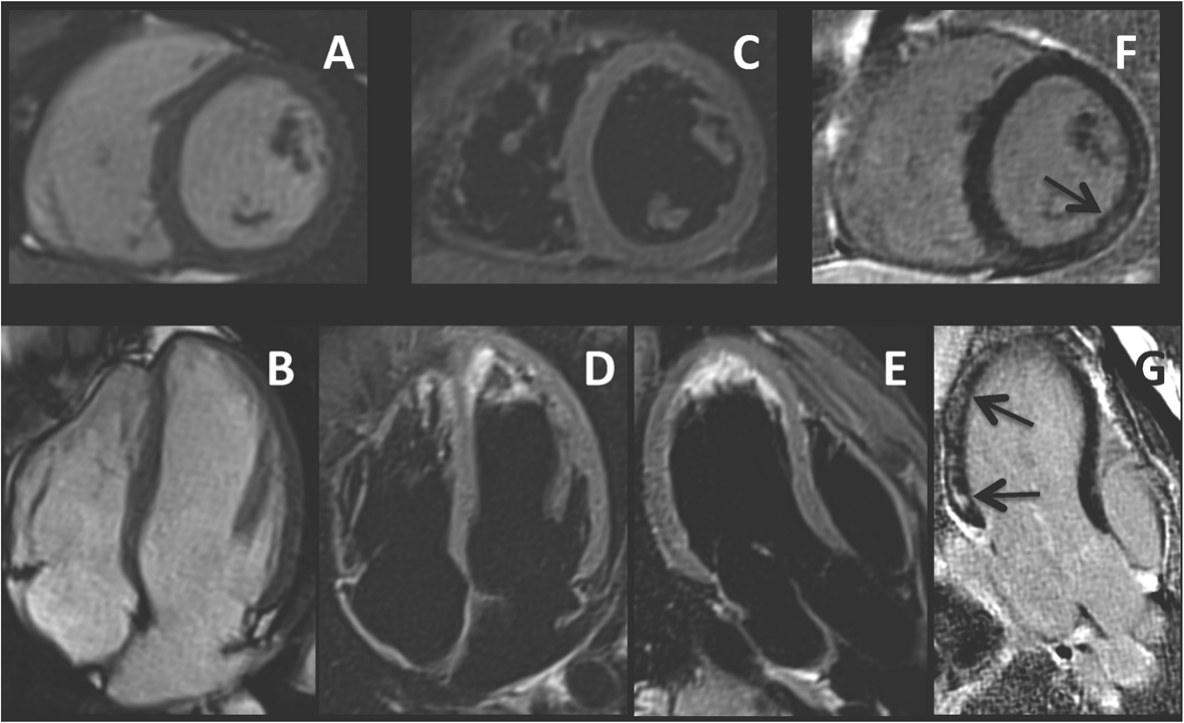
**Cine and late gadolinium enhancement (LGE) CMR images of the patient at baseline.** Panel **A** and **B**: cine (steady-state free precession sequence) CMR of short-axis **(A)** and 4-chamber **(B)** view showing normal left ventricular wall thickness. Panel **C**, **D** and **E**: T2-weighted short-TI inversion-recovery fast spin-echo image of short-axis **(C)**, 4-chamber **(D)** and 3-chamber **(E)** view showing the absence of myocardial oedema. Panel **F** and **G**: contrast-enhanced inversion recovery gradient echo image of short-axis **(F)** and 3-chamber **(G)** view showing LGE involving the inferolateral wall with intramyocardial distribution (black arrow).

**Figure 2:**
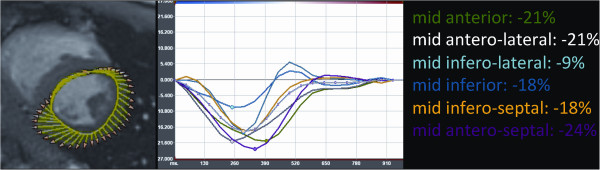
**Feature-tracking analysis.** 2D Cardiac Performance Analysis Software (TomTec, Munich, Germany) was used to measure LV deformation directly from cine CMR images (left panel). Feature-tracking analysis of cine LV mid short-axis slice demonstrated reduced circumferential strain of the mid infero-lateral wall (pale blue line in the right panel).

**Figure 3:**
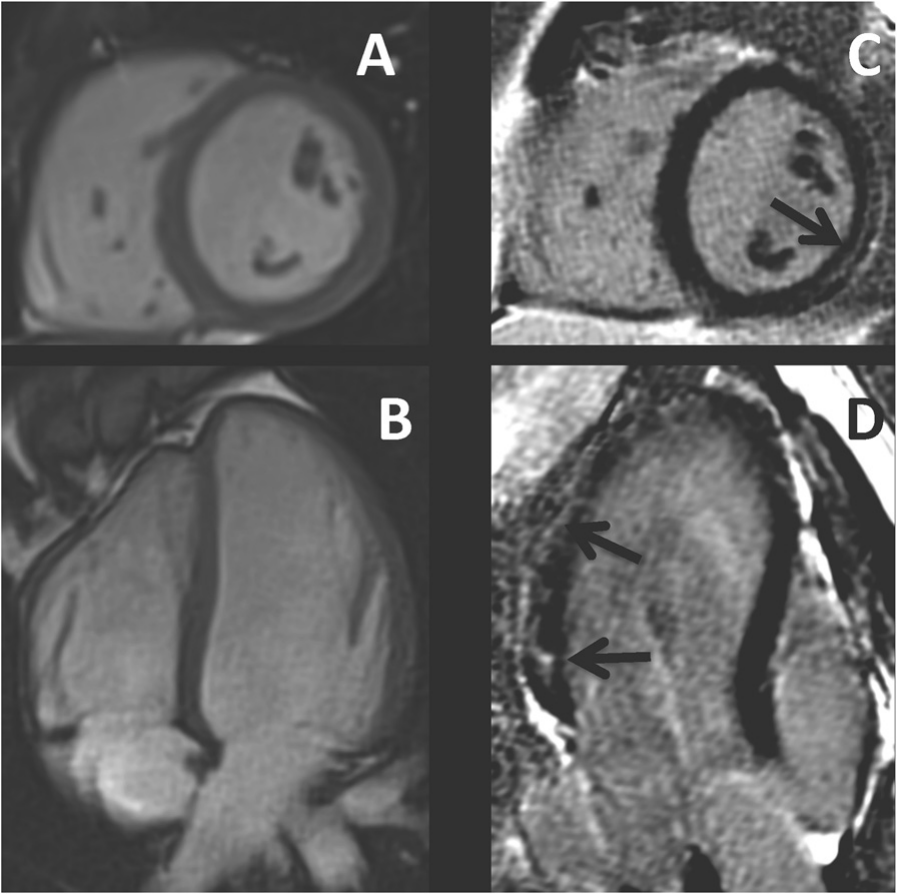
**Cine and late gadolinium enhancement (LGE) CMR images of the patient, acquired after 12 months of ERT.** Panel **A** and **B**: cine (steady-state free precession sequence) CMR of short-axis **(A)** and 4-chamber **(B)** view showing normal left ventricular wall thickness. Panel **C** and **D**: contrast-enhanced inversion recovery gradient echo image of short-axis **(C)** and 3-chamber **(D)** view showing LGE involving the inferolateral wall with intramyocardial distribution (black arrow), without significant changes from baseline examination (Figure 1).

A 19 years old boy was diagnosed with Fabry disease through a family-based molecular screening, demonstrating the presence of the R112H mutation in the *GLA* gene. The diagnosis was further confirmed by the assessment of enzymatic α-gal activity in leucocytes, which resulted undetectable. Subsequently, he was admitted for a global screening of disease clinical manifestations although, at the time of diagnosis, he appeared completely asymptomatic. His neurological and general examination, including cardiac auscultation, were unremarkable. Biochemical tests (including 24 h microalbuminuria, proteinuria and creatinine clearance), ophthalmologic assessment, audiometry, pulmonary function tests, brain and kidney magnetic resonance imaging did not show any abnormality.For the cardiac assessment he underwent first transthoracic echocardiography, 12-lead electrocardiogram, 24-hour Holter recording, and bicycle stress test, all resulting normal. Finally CMR with LGE imaging was performed; cine images (steady-state free precession) confirmed normal biventricular size and systolic function and normal LV wall thickness (Figure 1A-B). No signal alterations were detected on black-blood T1-weighted fast spin-echo imaging and T2-weighted short TI inversion recovery imaging (Figure 1C-E). Conversely, delayed enhancement imaging performed 10 minutes after injection of Gadolium chelate contrast agent showed LGE of the LV inferolateral wall with intramyocardial distribution (Figure 1F-G). Of note, despite the absence of LV wall motion abnormalities, feature-tracking analysis of cine LV mid short-axis slice demonstrated reduced circumferential strain of the mid inferolateral wall (Figure 2).Considering the male gender and the evidence of a cardiac involvement, ERT treatment with recombinant agalsidase alfa was started and administered intravenously at the standard dosage of 0.2 mg/kg every other week, without side effects. After 12 months of ERT cardiological assessment including ECG, echocardiography and CMR was repeated, showing no changes compared to baseline (Figure 3); in particular LGE of the LV inferolateral wall was still observed and unchanged (Figure 3C-D).

## Discussion

To our knowledge this is the first case reported so far, which demonstrates the presence of LGE without LV hypertrophy in a male affected by Fabry disease. Nevertheless, few CMR studies on Fabry patients have been published until now. Indeed, in the international consensus guidelines for Fabry disease, CMR is not included among the recommended exams for the basal evaluation and monitoring of cardiac complications and therefore it is not routinely performed [3,10,11].

In 2003 Moon and colleagues first reported CMR findings in 18 male and 8 female patients with Fabry disease, describing LGE in 9/18 males and 4/8 females. In 92% of patients LGE occurred in the basal inferolateral wall, with intramyocardial pattern. Two male patients with severe LV hypertrophy had additional hyperenhancement in other myocardial sites. One female patient with LGE had normal echocardiography [7].

In 2005 Weidemann and colleagues published a further cardiac imaging study (using echocardiography, strain rate imaging, and CMR) on 39 Fabry patients, finding LGE in 12 of them (2 females and 10 males). The inferolateral wall was confirmed as the typical LGE location. All subjects, of both genders, had also LV hypertrophy associated to LGE. All patients with LGE had severely reduced radial and longitudinal function. One patient with LGE died and histology confirmed fibrotic tissue in the inferolateral wall [8].

Recently, Niemann and colleagues published a large CMR study, including 104 patients (58 females and 46 males) with genetically proven Fabry disease. LGE was detected in 33% (n.19) of females and 48% (n.22) of males. Sixty-eight % of these patients showed LGE in the typical basal lateral location. Interestingly, 10 female patients without LV hypertrophy already presented LGE positive segments, while LGE was not found in male patients with LV thickness <; 12 mm. Based on these results, the authors concluded that cardiomyopathy disease progression in Fabry disease differs between male and female patients, only the latter showing fibrosis before hypertrophy [9].

On the contrary, our findings suggest that LGE can be the first sign of cardiomyopathy even in male patients. It is possible that LGE without LV hypertrophy has never been found in males affected by Fabry disease because only few patients have been studied with CMR until now and most of them, being older than the patient reported here (i.e. 46 ± 12 years in the study by Niemann), had already developed both LV hypertrophy and fibrosis at the time of the study. Since the proposed temporal sequence of Fabry cardiomyopathy (increasing wall thickness➔ replacement fibrosis) [8] is not always present even in men, further pathogenetic studies are needed to explain why fibrosis can progress independently from the development of hypertrophy in Fabry patients.

It is worth noting that our case, as well as 11 females already reported [9,7], did not manifest any cardiac symptom and had no wall motion abnormalities at the echocardiography but showed LGE at the CMR, suggesting that conventional assessment is not sufficient to detect subtle cardiac abnormalities. Our patient and 7/10 female with isolated LGE described by Niemann et al were diagnosed by genetic screening within the family after the identification of a proband. Taking into account these results, we should stress the importance to perform CMR to stage and monitor Fabry cardiomyopathy, after diagnosis in both genders, even in apparently asymptomatic patients with a normal echocardiogram. CMR can be also fundamental in the diagnostic process of patients evaluated for cardiomyopathy, as LGE in the midmyocardium of the basal-inferolateral wall seems to be characteristic of Fabry disease [12]. It should be acknowledged that LGE in the midmyocardium of the basal-inferolateral wall can also be observed in other pathologies, including previous myocarditis [13]. In our patient, however, history of symptoms suggestive of remote myocarditis was negative. Moreover, recently, non contrast T1 mapping by CMR has been proposed by Thompson et al. as a tool to distinguish LV hypertrophy do to Fabry disease, from other causes. In fact, septal T1 was found to be lower in Fabry patients compared with healthy controls and patients with similar patterns of LV hypertrophy, probably reflecting glycosphingolipids accumulation. The authors concluded that reduced non-contrast myocardial T1 values are the most sensitive and specific cardiovascular MRI parameter in patients with Fabry disease irrespective of sex and LV morphology and function [14]. Unfortunately this technique is not available at our hospital.

Of note, feature-tracking analysis of cine LV mid short-axis slice demonstrated reduced circumferential strain of myocardial segment presenting LGE, despite the absence of LV wall motion abnormalities; this underlines the utility of myocardial deformation imaging techniques (including speckle-tracking echocardiography, tagged-CMR and feature-tracking CMR) for the identification of subtle changes of myocardial function which cannot be detected with conventional measures such as qualitative wall motion assessment [15].

Cardiac fibrosis is considered a negative prognostic factor towards ERT response in patients who already show LV hypertrophy [16], and an increased amount of LGE (from 0.5 to 7.1%) after 12 months of ERT has been reported [17]. To date, no data are available on the effect of ERT in patients with isolated cardiac fibrosis. Our patient did not show any LGE change after one year of ERT treatment at the CMR assessment. Therefore, it is possible to hypothesise that cardiac fibrosis accompanied by LV hypertrophy would be a sign of a late stage cardiac disease, which rapidly progresses despite ERT. Instead, when cardiac fibrosis appears early in a non-hypertrophic myocardium, it may have a more favourable response to treatment. However, we do not know what would have been the natural course of fibrosis in our patient without ERT. Further CMR studies on large cohorts and long term follow up are needed to assess whether ERT initiated before the onset of LV hypertrophy can prevent the cardiomyopathy progression.

## Conclusions

The development of myocardial fibrosis does not necessarily require myocardial hypertrophy in both male and female patients with Fabry disease. CMR should be included in the routing diagnostic assessment and follow-up monitoring of all Fabry patients.

## Consent

Written informed consent was obtained from the patient for publication of this Case report and any accompanying images. A copy of the written consent is available for review by the Editor of this journal.

## Abbreviations

ERT: Enzyme replacement therapy; LV: Left ventricle; CMR: Cardiovascular magnetic resonance; LGE: Late gadolinium enhancement.

## Competing interests

The authors declare that they have no competing interests.

## Authors’ contributions

AS was the primary physician during patient’s first assessment and follow up, conceived the report and drafted the manuscript. GN provided cardiology supervision during scanning, and helped to draft the manuscript. GP assisted in image acquisition and interpretation and helped to draft the manuscript. AD performed the enzymatic and genetic tests and critically reviewed the manuscript. BB provided additional supervision and critically reviewed the manuscript. All authors read and approved the final manuscript.

## Pre-publication history

The pre-publication history for this paper can be accessed here:

http://www.biomedcentral.com/1471-2261/14/86/prepub
